# Multilingual Sentiment Analysis: State of the Art and Independent Comparison of Techniques

**DOI:** 10.1007/s12559-016-9415-7

**Published:** 2016-06-01

**Authors:** Kia Dashtipour, Soujanya Poria, Amir Hussain, Erik Cambria, Ahmad Y. A. Hawalah, Alexander Gelbukh, Qiang Zhou

**Affiliations:** 1 Department of Computing Science and Mathematics, University of Stirling, Stirling, FK9 4LA Scotland, UK; 2 Temasek Laboratory, Nanyang Technological University, Singapore, Singapore; 3 School of Computer Engineering, Nanyang Technological University, Singapore, Singapore; 4 Taibah University, Madina, Saudi Arabia; 5 CIC, Instituto Politécnico Nacional, 07738 Mexico City, Mexico; 6 Tsinghua University, Beijing, China

**Keywords:** Artificial intelligence, Natural language processing, Opinion mining, Sentic computing, Sentiment Analysis

## Abstract

With the advent of Internet, people actively express their opinions about products, services, events, political parties, etc., in social media, blogs, and website comments. The amount of research work on sentiment analysis is growing explosively. However, the majority of research efforts are devoted to English-language data, while a great share of information is available in other languages. We present a state-of-the-art review on multilingual sentiment analysis. More importantly, we compare our own implementation of existing approaches on common data. Precision observed in our experiments is typically lower than the one reported by the original authors, which we attribute to the lack of detail in the original presentation of those approaches. Thus, we compare the existing works by what they really offer to the reader, including whether they allow for accurate implementation and for reliable reproduction of the reported results.

## Introduction

With the growth of the World Wide Web, the amount of texts available online has been increasing exponentially. In particular, people express their opinions about different subjects and influence each other’s decisions by communicating their sentiments [56, 67]. The sentiment towards a brand on the Internet is important for any company concerned about the quality of its product, which makes it crucial for companies to understand people’s sentiments towards products and services [60]. The past few years have witnessed an explosion of commercial and research interest in the sentiment analysis field [4]. While information extraction techniques have been developed to deal with the ever-growing amount of texts in Internet, sentiment analysis has its own specific problems and difficulties [2]. Many approaches have been proposed to classify sentiments expressed in different channels such as Twitter, blogs and user comments.

**Fig. 1:**
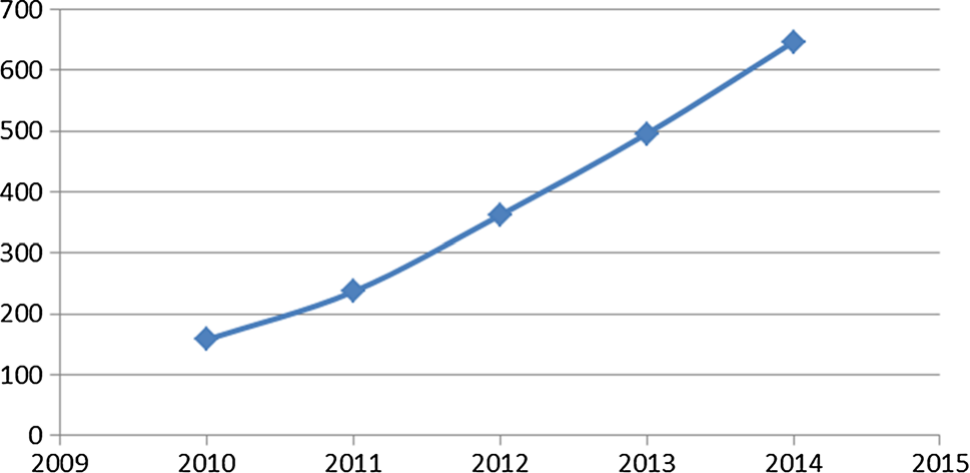
Number of publications on English sentiment analysis, per year [42]

**Fig. 2:**
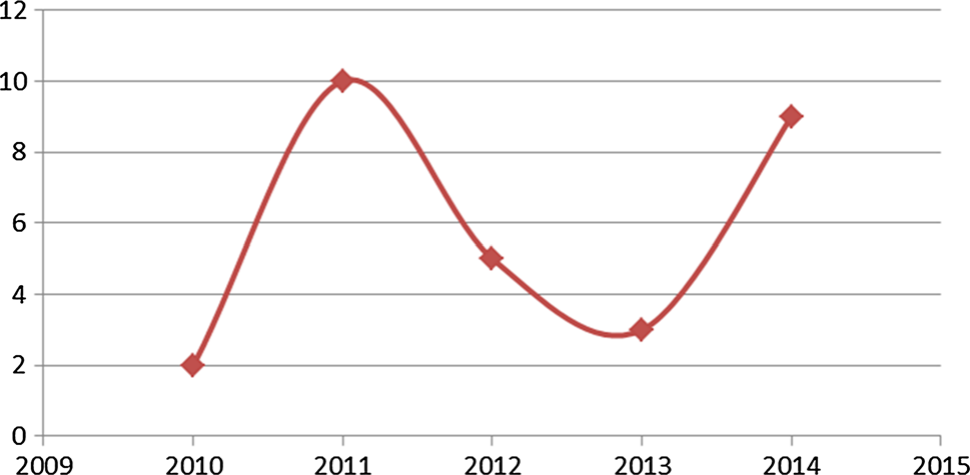
Number of publications on multilingual sentiment analysis, per year [28]

The majority of current sentiment analysis systems address a single language, usually English; see Figs. 1 and 2. However, with the growth of the Internet around the world, users write comments in different languages. Sentiment analysis in only single language increases the risks of missing essential information in texts written in other languages. In order to analyse data in different languages, multilingual sentiment analysis techniques have been developed [10]. With this, sentiment analysis frameworks and tools for different languages are being built.

One of the main problems in multilingual sentiment analysis is a significant lack of resources [4]. Thus, sentiment analysis in multiple languages is often addressed by transferring knowledge from resource-rich to resource-poor languages, because there are no resources available in other languages [18]. The majority of multilingual sentiment analysis systems employ English lexical resources such as SentiWordNet.

Another approach is to use a machine translation system to translate texts in other languages into English [18]: the text is translated from the original language into English, and then English-language resources such as SentiWordNet are employed [18]. Translation systems, however, have various problems, such as sparseness and noise in the data [4]. Sometimes the translation system does not translate essential parts of a text, which can cause serious problems, possibly reducing well-formed sentences to fragments [6].

Thus, researchers look for alternative approaches. The field of multilingual sentiment analysis is progressing very fast. In particular, multilingual lexical resources specific to sentiment analysis are being developed. For example, the NTCIR corpus of news articles in English, Chinese, and Japanese contains information on sentiment polarity and opinion holder for news related to the topics such as sport and politics [46]. However, sentiment analysis corpora and resources, even if created for multiple languages, cannot be used for other languages [33]. More research is required to improve results in the multilingual sentiment analysis discipline [20].

In this paper, we discuss existing approaches. More importantly, we report the results of our own experiments with these approaches on the same datasets, which allows direct comparison. For this, we have implemented eleven techniques following as closely as possible their descriptions in the original papers. Our results proved to be lower than the results reported by the original authors, which we attribute in the majority of cases to the lack of detail in their descriptions. Thus, in a way, we measured the real value of the information available on those approaches to the research community: a good approach poorly described is not useful for the community, even if it showed good results in its author’s own experiments, which are not available to the community. Thus, we evaluate what the original papers that we reviewed really offer to the reader, apart from only reporting the results their authors observed.

This paper is organized as follows. Section 2 briefly discusses multilingual sentiment analysis techniques and describes pre-processing, multilingual sentiment analysis resources, tools used in multilingual sentiment analysis, and the features used for machine learning. Sections 3, 4, and 5 present an overview of the state-of-the-art corpus-based, lexicon-based, and hybrid sentiment analysis techniques, correspondingly, both for English and for other languages. Section 6 gives a comparison of recently some of those methods in our own experiments on common datasets. Finally, Section 7 concludes the paper.

## Sentiment Analysis Framework

In this section, we will discuss the main general techniques used for sentiment analysis, as well as pre-processing procedures, lexical resources, tools, and features typically used in sentiment analysis systems.

### Main Techniques

Sentiment analysis systems can be classified into corpus-based approaches using machine learning, lexicon-based approaches, and hybrid approaches. Corpus-based methods use labelled data [70]; lexicon-based methods rely on lexicons and optionally on unlabelled data [57]; and hybrid methods are used based on both labelled data and lexicons, optionally with unlabelled data [51]. A sentiment lexicon is a collection of known sentiment terms [32].

### Pre-processing

The pre-processing task is an important step in multilingual sentiment analysis. It is used to remove irrelevant parts from the data, as well as to transform the text to facilitate its analysis.

#### Noise Removal

Usually the texts found in Internet have much noise such as HTML tags, scripts, and advertisements. Data pre-processing can reduce noise in the text and improve performance and accuracy of classification. The pre-processing step is crucial for multilingual sentiment analysis. The majority of the proposed approaches to multilingual sentiment analysis employ pre-processing of data to improve performance and accuracy.

#### Normalization

Often sentiment analysis and opinion mining is performed on texts from social networks and other user-generated contents. Such texts are characterized by very informal language, with grammar and lexicon that greatly differ from the usual language use, especially in Twitter. Such texts need to be transformed into a more grammatical form, more suitable for processing by natural language analysis tools. Such normalization is often performed using specialized lexicons, such as the multilingual Lexicon for pre-processing of social media, social networks, and Twitter texts developed by Posadas-Durán et al. [39] for English, Spanish, Dutch, and Italian.

#### Natural Language Analysis

The most important pre-processing tasks performed with natural language analysis techniques are tokenization, sentence splitting, stop-word removal, stemming, and part-of-speech tagging, among others. Tokenization is used to break the text down into words and symbols [14]. Sentence splitting is used to determine sentence boundaries. Stop words are common words in the given language that do not carry important meaning; their removal usually improves performance of sentiment analysis [41]. Stemming is a task used to transform words into their root form: for example, the word “working” is changed to its root form “work” [42].

### Sentiment Lexicons

Sentiment lexicons have been used in a number of approaches to multilingual sentiment analysis in order to improve the performance of classification. Sentiment lexicons are used mainly in lexicon-based sentiment analysis.

SenticNet is a lexical resource based on a new multi-disciplinary approach proposed by Cambria et al. [11] to identify, interpret, and process sentiment in the Internet. SenticNet is used for concept-level sentiment analysis. It is also used to evaluate texts basing on common-sense reasoning tools that require large inputs. However, it is not capable of analysing text with sufficient level of granularity. Sentic computing methodology is used, in particular, to evaluate texts at the page or sentence level. The purpose of SenticNet is to build a collection of concepts, including common-sense concepts, supplied with polarity labels, positive or negative. Unlike SentiWordNet, SenticNet does not assume that a concept can have neutral polarity. SenticNet includes a simple and clear API for its integration in software projects. It can be used with the Open Mind software. It guarantees high accuracy in polarity detection. Multilingual tools are available for SenticNet [64].

SentiWordNet is a lexical resource that assigns WordNet synsets to three categories: positive, negative, and neutral, using numerical scores ranging from 0.0 to 1.0 to indicate a degree to which the terms included in the synset belong to the corresponding category. SentiWordNet was built using quantitative analysis of glosses for synsets [52]. While SentiWordNet is an important resource for sentiment analysis, it contains much noise. In addition, it assigns polarity at the syntactic level, but it does not contain polarity information for phrases such as “getting angry” or “celebrate a party”, which correspond to concepts found in the text to express positive or negative opinions [11].

General Inquirer is a German lexicon supplied with positive and negative labels. For its construction, Google translate was used to translate words and terms into the German language; then, the words without any sentiment were removed from the lexicon. General Inquirer has been employed by Remus et al. [44]. The main advantage of General Inquirer is its widely used lexicon. Since it includes financial terms, it is used for financial sentiment analysis in the German language. However, its use is limited in other areas such as sport, politics, and product reviews [53].

SEL is a Spanish emotion lexicon that presents 2036 words supplied with the Probability Factor of Affective use (PFA) as the measure of their expression of basic emotions: joy, anger, fear, sadness, surprise, and disgust, on the scale of null, low, medium, or high. The lexicon was developed manually by 19 annotators, which had to agree above certain threshold for a label on the word to be included in the lexicon. The measure called Probability Factor of Affective use (PFA) was developed by the authors of this lexicon to incorporate agreement between annotators in decision-making on labelling the words: the greater the agreement, the stronger the expression of the emotion by the given word. The lexicon, freely available for download, has been used in opinion mining tasks on Spanish tweets [49].

### Sentiment Corpora

Lexical resources for sentiment analysis include, apart from sentiment lexicons, various corpora developed for sentiment analysis tasks. Sentiment corpora are used mainly for machine learning in corpus-based sentiment analysis.

YouTube dataset is a multimodal sentiment analysis dataset created by Morency et al. [35] from online social videos. In each clip included in the dataset, a person speaks in the camera expressing an opinion. The dataset has various characteristics challenging for sentiment analysis tasks, such as diversity, multimodal, and ambient noise. The topics discussed in online videos are very diverse. Diversity is important to analyse opinions: people express their opinions in different ways; some people express their opinions in subtle ways. The dataset provided age and gender information on the speakers, as well as topics of the opinions. In order to select best words to identify the sentiment of a sentence, multimodal techniques have been used. Since audio and video data have much noise, these data were recorded by using different cameras and microphones.

Explicit and implicit aspect corpora are used for aspect-based opinion mining. Hu and Liu [26] developed a corpus widely used in aspect-based sentiment analysis research. The original corpus contained data only for explicit aspect extraction, that is, for work with aspect words explicitly present in the sentence. Cruz-Garcia et al. [16] developed an implicit aspect corpus based on a subset of the corpus by Hu and Liu. In this new corpus, sentences are labelled with implicit aspects, i.e. aspects not named by any specific word in the sentence, and the corresponding implicit aspect indicators. This corpus, freely available for download, has been used in a number of research works.

MPQA is a subjective lexicon consisting of around eight thousand terms, which have been collected from different sources. The MPQA presents words supplied with part-of-speech tags and polarity (positive, negative or neutral), as well as intensity of polarity [59].

### Machine Learning Tools

WEKA, standing for Waikato Environment for Knowledge Analysis, is a freely available software package built in Java, which provides a large number of machine learning and data mining algorithms. The programme provides pre-processing and performance analysis data [25].

LIBSVM is a library implementing the support vector machine (LIBSVM) algorithm. It was built in 2000. The main purpose of LIBSVM is to help users to easily include SVM into their applications [13].

### Features Used

Machine learning features typically employed in sentiment analysis approaches include the following classes.

N-grams represent continuous sequences of *n* items in the text. The n-grams of size one are called unigrams, those of size two are called bigrams, and those of size three are called trigrams. For example, in the sentence “I went to the cinema”, the bigrams (after removing the stop-word “the”) are “I went”, “went to”, “to cinema”, and the trigrams are “I went to” and “went to cinema” [40].

Document frequency is the total number of documents in the dataset that contain a given word. A threshold is calculated for document frequency of words in the training corpus, and the words with document frequency lower than some threshold or higher than another threshold are removed at the pre-processing stage. This process is important for term selection. Tt is used to scale large datasets to reduce the computation cost of their processing.

Term frequency (TF) is the number of occurrences of an item (such as a word or n-gram) in a given document. It is often used in combination with inverse document frequency (logarithm of the inverse of the share of the documents in the collection that contain the given term) in the form of the TF-IDF feature.

Mutual information (MI) is used to measure the dependence between two different variables [36]. Mutual information is used in statistical language modelling [68].

Information gain (IG) measures goodness of features in machine learning. It is used to measure the amount of information contributed the classification process by the absence or presence of a term in the document [68].

Chi-square test is used to calculate the category of terms [68]. Chi test measures the divergence from expected distribution based on the features that are independent from the class value [58].

## Corpus-Based Techniques

In this and the next sections, we will discuss the state-of-the-art approaches to sentiment analysis classified into corpus-based, lexicon-based, and hybrid ones, for both English language and other languages. In particular, in this section we present corpus-based techniques, development of which focuses on feature engineering and model selection. The majority of the techniques presented here use annotated corpus and machine learning models to train a suitable sentiment analysis classifier.

### English

Shi and Li [47] developed a supervised machine learning technique for sentiment analysis of online hotel reviews in English by using unigrams features. They used features such as term frequency and TF-IDF to identify the document polarity as positive or negative. The data were separated into training and testing sets with different data instances. The instances in the training set covered the target values. The support vector machine (SVM) has been used to develop a model able to predict target values of data instances [47]. The SVM classifier has been chosen because it has been reported to perform better than other classifiers [38], though Tong and Koller [55] consider Naive Bayes and SVM the most effective classifiers among machine learning techniques [61]. The hotel-review corpus contained 4000 (positive and negative) reviews; the reviews have been pre-processed and tagged as positive and negative. Then, the obtained sentiment classification model has been used to classify live information flow into positive and negative documents. The TF-IDF feature performed better than simple term frequency [47].

Another study [10] used supervised classification for identification of the sentiment in documents. They applied their method to sentences found in Internet, in particular, in blogs, forums, and reviews. The features of the sentences were extracted using a state-of-the-art algorithm. Sentence parsing has been used for a deeper level of analysis. Finally, the method of active learning has been used to reduce workload in annotation [15]. After the pre-processing stage, there were different features selected, such as unigrams, stems, negation, and discourse features. The SVM, Maximum Entropy, and multimodal Naïve Bayes classifiers have been employed as machine learning algorithms. For linearly separable data, SVM gives classification results with minimal error. The multimodal Naïve Bayes classifier is very simple to use for efficient classification and with incremental learning [31]. The Maximum Entropy classifier is efficient in extracting information that leads to good results [7]. English-language corpora were collected from blogs, reviews, and forum sites such as www.livejournal.com or www.skyrock.com.

The Maximum Entropy classifier showed 83 % accuracy, which is better compared to other classifiers used in this study, namely SVM and multinomial Naïve Bayes; however, other approaches [47] used SVM to evaluate datasets, and other machine learning techniques have been reported to have accuracy lower than that of SVM.

The main advantage of this approach is that it involves less building effort and is simple to develop. A disadvantage of this approach is the lack of high-quality training data, because data collected from blogs contain many grammatical errors, which negatively affect classification performance [10].

### Other Languages

Habernal et al. [23] proposed an approach for supervised sentiment analysis in social media for the Czech language. Three different datasets have been employed; first dataset was collected from Facebook, basing on top comments in popular Czech Facebook pages. The Facebook dataset contained positive, negative, neutral, and bipolar information. The second dataset was a movie review dataset downloaded from a Czech movie database. The third dataset contained product review information collected from large online Czech shops. After the data pre-processing step, the n-gram feature has been extracted. The unigrams and bigrams were used as binary features. In addition, the minimum number of occurrences of character n-grams has been established. Part-of-speech (POS) tagging provided characteristics of specific posts. Various POS features have been used, such as adjectives, verbs, and nouns. Two different emoticon lists have been used: one for positive and one for negative sentiment. Another feature used was Delta TF-IDF, a binary word feature, which showed good performance. Delta TF-IDF uses TF-IDF for words, but it treats words as positive or negative.

To evaluate the dataset, two different classifiers were trained: SVM and a Maximum Entropy classifier. The F-measure for combination of features such as bigrams, unigrams, and emoticons was 0.69. The emphasis of this approach was on feature selection. The features that were selected were bigrams, unigrams, POS, and character n-grams. This approach is useful for sentiment analysis in Czech social media. However, it cannot be directly used for other languages, and its results are not very helpful even for Czech social media. Still it can help researchers extend sentiment analysis methods to the Czech language [23].

Tan and Zhang [54] introduced an approach for sentiment classification for the Chinese language. First, POS tagging was used; the aim of using POS tagging was to parse and tag the Chinese text. After POS tagging, feature selection was used to determine discriminative terms for classification. Finally, a machine learning approach was used for sentiment classification. Feature selection included four types of information: document frequency, Chi-square feature, mutual information, and information gain. The threshold was defined for the document frequency of words and phrases in the training corpus, and the words with the document frequency lower than a predefined threshold or higher than another predefined threshold were removed. In order to calculate the association between terms, CHI was used. Mutual information was used for statistical language modelling. Information gain measures the amount of information useful for prediction of the category that is contributed by the presence or absence of a given term in the document.

There are various datasets available online for use in Chinese sentiment classification. The Chinese sentiment corpus ChnSentiCorp, collected from online documents, is an online benchmark sentiment analysis database. It includes 1021 documents in three domains: education, movies, and house. For each of these domains, there are positive and negative documents. The centroid classifier, SVM, Naïve Bayes, *k*-nearest neighbour classifier, and winnow classifier were compared. The overall accuracy of the SVM classifier was better than that of other classifiers.

This approach is unique in comparison with other approaches in that the feature selection scheme is different. The features that are selected are document frequency, mutual information, Chi-square statistic measure, and information gain. Other approaches usually employ such features as bigrams and unigrams. The results of this approach show that of such features as information gain, document frequency, Chi-square statistics, and mutual information, information gain is the best feature and can be recommended for future applications. The main disadvantage of this approach is use of traditional features such as Chi-square statistics, document frequency, and mutual information [54].

Ghorbel and Jacot [21] proposed an approach for sentiment analysis of French movie reviews. Their method relies on three types of features, namely lexical, morpho-syntactic, and semantic features. The unigrams were selected as a feature. The goal of this system was to find polarity of the words. The part-of-speech tags were employed to augment unigrams with morpho-syntactic information, in order to reduce word sense ambiguity and to control negation before polarity extraction. SentiWordNet was used to determine polarity of words. This information was used to measure the overall polarity score of the review [52]. SentiWordNet is an English-language resource; in order to use SentiWordNet, French reviews were translated into English before extraction of polarity. The words were lemmatized before looking them up in a bilingual dictionary; then part-of-speech tags were used for sense selection, to remove uncertain senses, and to predict the correct synset. The dataset of French movie reviews contained 2000 documents: 1000 positive and 1000 negative reviews of ten movies.

The SVM classifier was used for classification. The overall performance on French movie reviews using unigrams, lemmatization, and negation was 92.50 % for positive reviews and 94 % for negative reviews. This approach combined lexical, morpho-syntactic, and semantic orientation of words to improve the results. The accuracy was improved by 0.25 %. The semantic orientation of the words was extracted from SentiWordNet, which further improved the result by 1.75 %.

A disadvantage of this approach is that words need to be translated into English prior to use SentiWordNet, which is an English-language resource. The quality of translation had a negative effect on the performance of the classifier, since translation of words does not preserve the semantic orientation due to differences between languages [21].

Balahur and Turchi [5] introduced a hybrid technique for sentiment analysis of Twitter texts. The sentiment analysis tools for various languages were developed to minimize the effort to produce linguistic resources for each of these languages; research on the use of machine translation systems to produce multilingual data was conducted in the context of Twitter texts.

The pre-processing was employed to normalize the texts: at this phase, the linguistic peculiarities of tweets were taken into consideration. Spelling variants, slang, special punctuation, and sentiment-bearing words from the training data were substituted by unique labels. For example, the sentence “I love car” was changed to “I like car”; according to the General Inquirer dictionary, *love* and *like* both have positive sentiment.

This approach can be used for various languages with minimal linguistic processing. Only tokenization was used; the method does not require any further processing. The final system should work similarly for all languages.

A standard news translation system was used to obtain data in various languages such as Italian, German, Spanish, and French. The original dictionary was created based on translation of English and Spanish texts into a third language. The dictionary was created for fifteen different languages. This approach includes two main stages: the pre-processing step and the application of a supervised machine learning technique. Support vector machine sequential minimal optimization (SVM SMO) was employed to identify features such as n-grams and bigrams in the training data [5].

The accuracy on English language was higher than on other languages. The main novelty of this approach was the pre-processing step. The pre-processing of Twitter texts is very important for sentiment analysis, and it significantly affects the accuracy of the classifier. The normalization of tweets at the pre-processing step can improve the accuracy. The main disadvantage of this approach is that on English language better accuracy was obtained in comparison with other languages, while on other languages such as Spanish and Italian the approach did not perform well [5].

Duwairi and Qarqaz [19] introduced a supervised technique for sentiment analysis of Arabic tweets. The authors generated a dataset using 10,000 tweets and 500 Facebook reviews in various domains such as news and sport. A number of pre-processing techniques were used in this study including removing duplicated tweets, empty tweets, and emoticon-only reviews. In order to determine the sentiment of collected tweets and Facebook reviews, a number of volunteers were asked to label each tweet or comment as positive, negative, neutral, or other.

A number of pre-processing steps such as tokenization, stemming, forming bi-grams, and detection of negation were then applied to the tweets and Facebook comments. Finally, three supervised machine learning techniques were applied on the prepared dataset, namely *k*-nearest-neighbour, Naïve Bayes, and SVM classifiers. The tenfold cross-validation method was used for evaluation. It showed that SVM outperformed both *k*-nearest-neighbour and Naïve Bayes classifiers. A limitation of this study was that the number of trained data was rather small.

## Lexicon-Based Techniques

The development of lexicon-based techniques mainly focuses on the different semantic orientation methods. Such techniques use different lexicon resources for sentiment inference.

### English

The unsupervised semantic orientation (SO-PMI-IR) method has been proposed for the sentiment classification of movie reviews. In the semantic orientation, text is classified basing on the score of the chosen sentences. The pointwise mutual (PMI) information for extracted features is calculated as

$${\text{PMI}} \left( {t, c} \right) = \log \frac{{p \left( {t,c} \right)}}{p \left( t \right) p\left( c \right) }.$$

Here, *c* denotes the category and *t* indicates the term [69]. Pointwise mutual information is used to measure the degree of compatibility of a term and category [66].

**Fig. 3:**
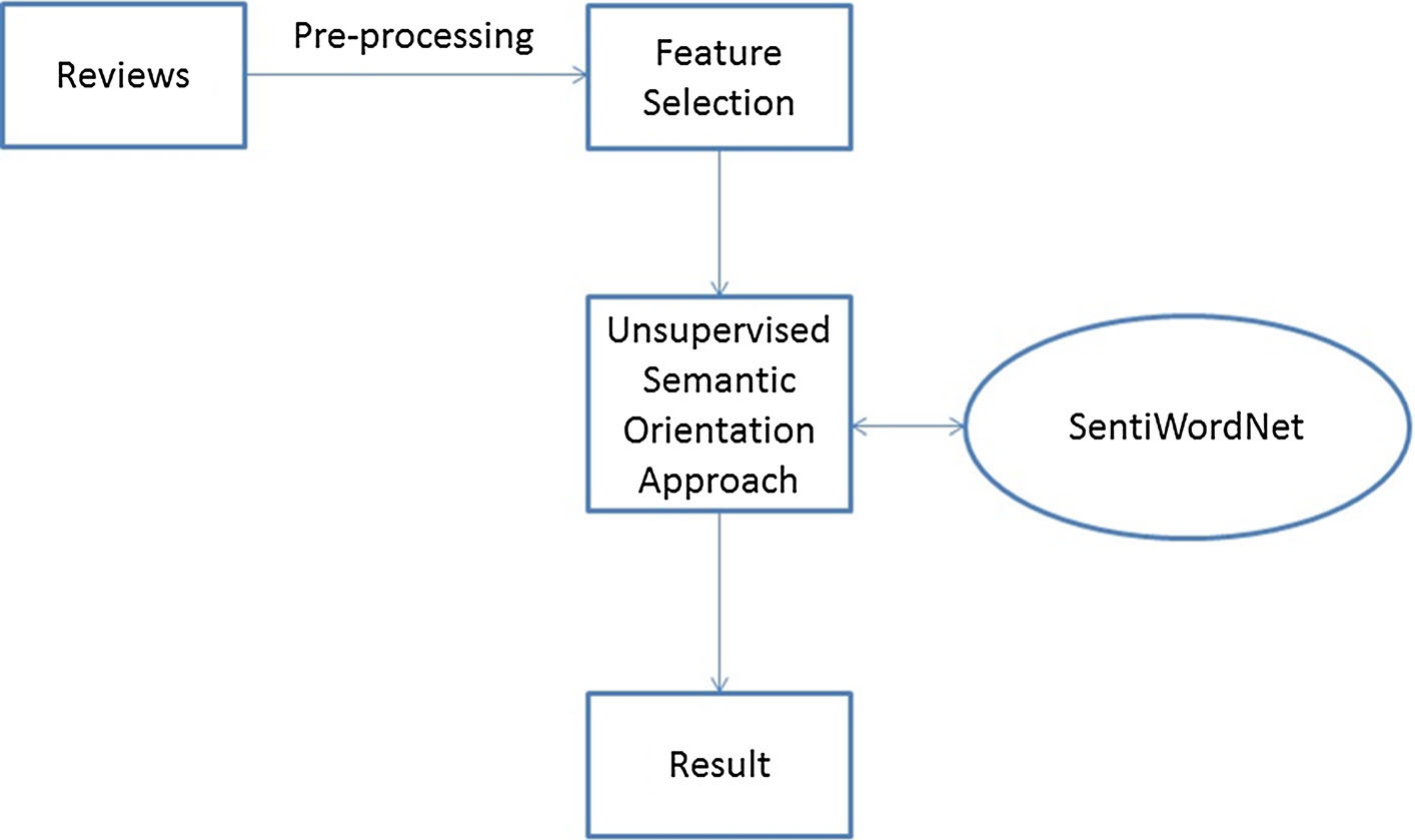
Flowchart of the approach of [52]

Singh et al. [52] used the unsupervised semantic orientation with part-of-speech tagging on the Cornell movie review dataset; this approach showed the best results in our own evaluation; see Sect. 6.1. Feature extraction was done for all reviews. The semantic orientation was calculated for reviews; then adjectives were extracted and the semantic orientation value was assigned to them. Aggregation was done for semantic orientation: each positive term +1 was added to the total document score and for each negative term, –1. Thus, the semantic orientation of each review was the total semantic orientation values for the extracted terms. Then, a threshold of 5 on the absolute value of the score was used to classify a document as positive or negative basing on the aggregation score. This approach was based on SentiWordNet. The features were extracted, and then SentiWordNet was employed to check the scores for the selected features. SentiWordNet provides scores from 0.0 to 1.0 [11]. Two different datasets were used; one dataset contained one thousand positive and one thousand negative reviews, and another dataset contained seven hundred positive and seven hundred negative reviews. Figure 3 presents the main steps of this approach.

This approach can be easily extended to other languages. In particular, it detects multiword expressions and can handle sarcasm; some languages, such as Persian language, make heavy use of multiword expressions and sarcasm [45]. In the future, this approach can be improved if different dialects can be detected; for example, Persian language has many different dialects [45], as do many other languages, such as Arabic, German, and Chinese.

The main disadvantage of this approach was that it required computationally expensive calculation of PMI, which was very time consuming [52]. The use of PMI in this approach did not improve the performance, which was still below that of other machine learning methods [43].

In another research, a method for unsupervised sentence classification of product reviews by using tools such as SentiWordNet was introduced. This method consisted of six steps. The first step was to collect different online reviews. The second step was the pre-processing of the reviews. The third step was building lists containing noun features and extracting the noun phrases. The fourth step was to classify sentences into objective and subjective sentences. The fifth step was the opinion sentence detection that calculated the semantic orientation of words related to the weight of the word in the SentiWordNet dictionary. Finally, the last step was to calculate the weight for each sentence and review and determine its polarity. This method obtained regular accuracy. The dataset that was used for evaluation contained online reviews of cameras such as Canon and Nikon. After data collection and pre-processing, the sentences were classified into objective and subjective types. To find semantic orientation of subjective sentences, SentiWordNet was used. The final semantic score was calculated to identify positive and negative statements. However, in other approaches, such as that by Singh et al. [52], data pre-processing consisted of part-of-speech tagging, the sentences were not classified into objective or subjective types, and an aggregation procedure was used to calculate the semantic orientation score [22].

The main disadvantage of this approach was the use of SentiWordNet. Its results show that SentiWordNet was ineffective in discovering sentiment words and performing the classification task [8].

### Other Languages

Wan [57] proposed an approach to leverage English resources to increase performance of Chinese sentiment analysis. The approach included various stages. First, a translation system has been used to translate the Chinese reviews into English. There were various translation systems used, such as Yahoo and Google, to translate Chinese reviews into English. After translation, the semantic orientated approach has been used to calculate the value of reviews. This approach used negation lexicon to reverse the semantic polarity of the words or phrases changing the value of the term to positive or negative. The unsupervised method was very simple. It used positive and negative lexicons; negation lexicon contained different terms used to reverse the semantic polarity of specific terms; intensifier lexicon consisted of words and phrases able to change the degree for the term to positive or negative.

In order to evaluate the performance of the introduced method, one thousand product reviews were collected for Chinese IT products such as mp3 players, mobile phones, laptops, and cameras. Chinese reviews were translated into English and analysed in both languages to obtain better accuracy. The results showed an overall performance improvement. This approach employed the ensemble to improve performance of the classification by 0.25 % [57].

The advantage of this work was in comparing different translation systems and determining the best system that can be used for future research. A disadvantage of this approach was in that translation of the reviews had a negative effect on performance [57].

Carroll [12] developed an innovative unsupervised model for the Chinese product reviews. The approach used comprehensive semantic analysis of words in the Chinese language. Lexical items were sequences of Chinese characters, ignoring punctuation marks. Each zone was classified as positive or negative. The iterative process was able to increase the seed vocabulary into broad vocabulary that consisted of a list of sentiment-bearing lexical item. A classifier was run on Chinese product reviews, giving as outcome positive and negative documents. The sentiment density has been calculated as a proportion of opinion zones in the documents. The sentiment density was not an absolute value, but it was used to compare documents with each other. The sentiment density of 0.5 does not mean half-opinionated document; it can be interpreted as indicating that the review is less opinionated than a review with density of 0.9. The classifier was able to reach 87 % F-measure for sentiment polarity [12]. A disadvantage of this approach was in using a corpus that did not help to detect the polarity of the words [12].

Zagibalov and Carroll [71] used automating seed words for selection in the Chinese language. In unsupervised learning, the training data need not be annotated. The approach did not require word segmentation. The lexical items lexicon was used to treat Chinese characters. In order to improve the classifier to find the seeds automatically, two assumptions have been used: the first assumption was that the attitude was stated by using negation of word items with their opposite meaning; this assumption was used to find negative lexical items from positive seeds. The second assumption concerned polarity of seeds that needed to be identified. To identify the polarity of a seed word, the lexicon was used to reach gold standard for positive lexical item. The sentiment classification and iterative technique were used in the unsupervised method. The method was used to find seeds automatically from raw text. To find positive seeds from the corpus, a special algorithm was developed. It operated over the sequence of characters that should be checked for containing negation or adverbials. This method does not use pre-segmentation or grammar analysis; the unit of processing is a lexical item. Input sequences of Chinese characters did not include punctuation marks and zones. A single zone was classified either as positive or negative, and the corresponding scores were calculated. Then, iterative retaining was used to increase the seed vocabulary in the list of sentiment-bearing lexical items. The latest version of the classifier was used on the corpus to classify documents as positive and negative. The iterative retaining was stopped when there was no modification to the classification of the document. To test the method on the dataset obtained from Chinese product reviews website, the reviews were tagged by polarity and the duplicate reviews were removed.

The main difference of this approach is the seed corpus. To develop the seed corpus, the following algorithm was used:

- The sequence of characters should be delimited by non-character symbols;
- The number of occurrences of a sequence that follow negated adverbial was counted;
- The number of occurrences of a sequence without such construction was counted;
- All such sequences were found.

A disadvantage of this approach is that it is very difficult to build and requires extensive parameter tuning [24].

Zhang et al. [72] presented a lexicon-based approach for classification of Chinese reviews of different products. This Internet-based method (PMI-IR) consisted in four phases. The first phase was parsing and POS tagging of the reviews; the second phase was extraction of two phrases conforming to a specific pattern in part-of-speech tags; the third phase was to identify phrases and calculate the semantic orientation of SO for all extracted phrases in the reviews. The approach contained different phases that were after the data pre-processing step: the sentiment expression was extracted from the Chinese review, snippet was formed, sentiment orientation of the expression was determined, and finally, sentiment classification for Chinese review was performed. This approach used snippets to identify the sentiment polarity of the phrases. A snippet is a small text from the documents, and it is located below the links returned by search engines. A snippet contains part of query words and allows previewing the query words in the documents. The PMI-IR algorithm was used to calculate the semantic orientation; the words have been estimated by using returned snippets. For example, to calculate the polarity for the word “poor”, the query has been sent to Google and returned snippets were crawled.

In order to evaluate the approach, a mobile phone review dataset, of forty positive and forty negative reviews, was used. The main difference of this approach is the use of snippets. Other approaches usually used online reviews, blogs, and Twitter texts.

Al-Ayyoub et al. [3] proposed an unsupervised approach to sentiment analysis of Arabic tweets. This approach included two stages: The first stage was collecting and pre-processing the tweets. The pre-processing step included stop-word removal and stemming. The second stage was the development of a sentiment lexicon, with the sentiment scores in the range between zero and one hundred. Scores from zero to forty corresponded to negative sentiment, forty to sixty to neutral, and sixty to one hundred to positive. These values were combined with each other to calculate the sentiment value of the sentence. The overall accuracy of this approach was 86.89 %. A disadvantage of this approach is that it is not able to handle different Arabic dialects [3].

## Hybrid Techniques

In this section, we present resource-hybrid techniques, which combine corpus-based and lexicon-based approaches, focusing on the domain adaption of sentiment analysis for the resource-poor languages or special domains. These techniques mostly use both annotated corpora and lexicon resources for learning more useful sentiment analysis resources.

### English

Mizumoto et al. [34] introduced unsupervised approach to identify sentiment polarity of the stock market. The polarity of the sentiment for stock news market was identified by using a polarity dictionary that contained words and their polarities. In this method, for a small amount of words, polarity was determined manually. The polarity of new words was then identified automatically. The new dictionary method has been built for unlabelled news. The dictionary contained a small number of words with their polarities such as positive and negative words. If a word was situated in one sentence with both positive and negative words, the co-occurrence of frequency for negative and positive polarity was calculated. The bias of co-occurrence was measured; most of the words were occurring with positive and negative polarities; the rate of co-occurrence of positive and negative polarity of dictionary has been used; then the polarity of those words that were not added was estimated. Finally, the polarity of words was determined. Two different thresholds were introduced, namely thresholdP and thresholdN. The thersholdP value was used to add words to the positive polarity dictionary, and thresholdN was used to add words to the negative polarity dictionary. The threshold values varied from 0.5 to 1. Words with occurrence frequency lower than ten were excluded as not reliable.

An online stock market news dataset has been used for evaluation. It contained 62,478 news items. A polarity dictionary was built automatically with a semi-supervised technique. The method assigned 45 % of correct polarity values for all news items.

The main difference of this approach compared to the supervised and unsupervised learning was in using the bootstrapping approach. The bootstrapping approach is a statistical technique consisting in a very simple procedure based on computer calculations. This approach was used for semi-supervised learning, because it used small amount of labelled data and large amount of unlabelled data [34].

### Other Languages

Zhu et al. [73] developed a semi-supervised method based on bootstrapping to analyse microblog data. An SVM classifier was trained to classify items as subjective or objective and for polarity classification. The bootstrapping method was automatic classification. This method used a small labelled dataset. Using a corpus with training data, unlabelled data were labelled by the classifier. If a part of samples was integrated into training corpus, bootstrapping can obtain classifier with some labelled data and a large amount of unlabelled data. The features that were selected contained effective characteristics such as word, part-of-speech tags, and emoticon symbols. In order to improve performance, the emoticons have been divided into positive and negative via emoticon lists. The probability to be positive or negative for emoticons was calculated. SVM with default parameters was used for classification of the polarity. The Chinese microblog content was used as a dataset. It was difficult for sentiment analysis because the expression was random. The main problem of this approach was that its accuracy was low. This approach selected different features such as specific symbols and microblogs emoticons set.

Remus et al. [44] proposed a new approach for semi-supervised German-language sentiment polarity classification. The proposed system was called SentiWS; the dictionary that was used in the SentiWS is freely available online. The weight of entry expression of polarity between –1.0 and +1.0 was calculated. The final stage was to evaluate the performance and accuracy. The part-of-speech tagging was used to build the dictionary, which included positive adverbs, negative adverbs, positive adjectives, negative adjectives, positive nouns, negative nouns, positive verbs, and negative verbs. The SentiWS used several resources to supply words with their semantic orientation. The first resource was the General Inquirer lexicon using Google translator to categorize positive and negative expressions semi-automatically in the German language. The reason for using General Inquirer was that it was widely accepted. The second resource was co-occurrence analysis of rated reviews. The rated reviews can be tagged from strong positive to strong negative. The co-occurrence is important for domain-dependent terminology. The third resource was the German Collocation Dictionary. This dictionary was able to group words that were collated, which were nouns classified by semantic similarity [17, 27, 63]. The German collation dictionary contains 25,288 semantic groups. The pointwise mutual information has been used to calculate the weight of the polarity. The purpose of using pointwise mutual information was to find semantic information from semantic association.

In order to evaluate the method, 2000 sentences were selected from a corpus and manually divided into positive, negative, and neutral. This approach used the General Inquirer lexicon that was not used in other approaches. General Inquirer includes words categorized into positive and negative. Since it has been translated, the translation process may have affected the quality of the process.

 This approach contains suffered from missing and ambiguous words, which had a negative effect on the performance [44].

Guan and Yang [29] proposed a technique for sentiment analysis in Chinese microblogs in order to develop an approach in analysis of characters for Chinese microblogs compared to traditional online media such as blogs. The purpose of this study was to classify opinion in microblogs into positive or negative. The method required a pre-processing step such as word segmentation and noise symbol filtering. The classification features needed to be extracted for every individual message, and finally, self-training was used to classify the unlabelled data. One of the methods for the semi-supervised learning is self-training, where labelled and unlabelled data together are used as a training corpus. Self-training is a wrapper algorithm that is used in the supervised methods. First, it begins with training labelled data; when the iterations start, it is able to determine unlabelled data that exist with labelled data. The overall performance of the self-training sentiment classification for Chinese is not good compared with supervised learning methods. Reverse self-training is a method that has been used for selecting strategy in labelled and unlabelled learning. The performance can be improved if some of the samples, where the classifier detects low certainty for associated polarity, are labelled. The technique used in the reverse self-training is simple: the classifier determines the unlabelled data, reverses data, and finally adds the most confident unlabelled data and less confident reverse data to the training set. Once this process is completed, the classifier is able to cover the decision space without many majority class samples.

For the evaluation of the Chinese microblogs, the NLP and CC2012 datasets have been employed. They contain twenty topics, 2207 subjective, 407 positive, and 1766 negative items. The sentiment lexicon has been used, provided by HowNet that contains 836 positive sentiment words and 1254 negative sentiment words. The precision for self-training was 0.895, recall was 0.667, and F-measure was 0.765. The precision for reversed self-training was 0.919, recall was 0.683, and F-measure was 0.784.

The main difference of this approach from previous approaches was in using specific domain, such as digital product reviews. The sentiment classification of microblogs contains multi-domain information. The performance of trained model of domain can be very poor when it shifts to another domain.

Mahyoub et al. [30] proposed an approach for determining sentiment for Arabic text. This study presented a semi-supervised approach to identify Arabic text sentiment by creating an Arabic sentiment lexicon that was able to assign sentiment scores for Arabic words. The Arabic sentiment lexicon was created using the Arabic WordNet. The authors used a small positive and negative Arabic wordlist as a training set, and the main goal was to use it to determine the polarity of all other words in Arabic WordNet. They proposed a semi-supervised algorithm that used the relations between the Arabic WordNet words to spread the sentiment score. The scores in this study were similar to the SentiWordNet ones: a word could be positive, negative, or neutral. The main difference was in that the score was not normalized to be between 0.0 and 1.0. In total, 7500 words were processed, and about 6000 of these words were found to be neutral, while 800 words were found to be positive, and 600 to be negative. The constructed Arabic sentiment lexicon was evaluated using a number of Arabic sentiment corpora, namely the OCA corpus, which contains movie reviews and a book review corpus. A machine learning classifier was applied using both vector space model [62] and Naïve Bayes model. The technique achieved 96 % classification accuracy. However, its limitation was that most of the Arabic reviews and tweets contained informal words, as well as words in different dialects and special regional words that have not been considered in this study.

## Comparison of Multilingual Sentiment Analysis Techniques

In the previous sections, we have described a variety of sentiment analysis techniques. For practical applications and for research work, one would need to choose the best performing approaches. However, direct comparison between those systems is difficult due to a number of factors. First, the original authors report the results on very different datasets, which makes comparison between the reported figures not fair. More importantly, the original authors describe their systems with varying degree of detail and accuracy, which makes the reported results not always reproducible. With this, even if a method showed excellent results in the authors’ own evaluation, lack of detail in their publication may render it unusable in practice for the readers.

To address these two difficulties, we implemented the methods reported in the papers discussed above and applied them to two datasets. In our implementation, we did our best to follow as exactly as possible the descriptions in the respective papers; however, in some cases due to lack of explanations, we had to guess what the authors meant, or had to omit parts of the method when the original paper gave too little clue as to what was meant to be done. For example, Tan and Zhang [54] mentioned that they implemented four traditional feature selection methods, but did not provide any details on how they were implemented; we had to implement some feature selection approach, which might not coincide with the one used by Tan and Zhang [54]. Similarly, the original authors often did not specify the tools they used to implement their approaches; in our experiments, we used Java and Python.

With this, our quantitative comparison reflects not the value of the methods as known only to their authors and implemented on their own computers not accessible by anybody else, but the real value of the information on those methods available to the research community through the respective publications—which, unfortunately, is far too often not the same.

In such uniform implementation, we also observed advantages and disadvantages of the methods, such as simplicity of implementation and extensibility, which allowed for qualitative comparison of the methods.

We realize, however, that comparison of approaches on a common dataset may not be fair to the approaches designed for a specific application domain. For example, the system by Shi and Li [47] was designed for a hotel reviews dataset, which can explain why in our experiments its performance was much lower than the one reported by its authors.

### Quantitative Comparison on Common Data

We evaluated the performance of a number of existing multilingual sentiment analysis approaches on two popular datasets that reflect two important application domains of sentiment analysis: a movie review dataset and a product review dataset. As the movie reviews dataset, we used the Cornell movie review data [37], which contains 1000 reviews labelled as positive and 1000 labelled as negative. As the product reviews dataset, we used the Blitzer dataset [9], which contains Amazon product reviews. Specifically, we used the reviews on books and DVDs. These datasets, publicly available online, are most commonly used by researches [37]. On the other hand, these datasets are different enough to test the methods on robustness.

We implemented existing approaches using various tools and programming languages, such as LibSVM, WEKA, Java, and Python. The results of our evaluation of the selected multilingual sentiment analysis approaches are shown in Table 1. The table shows the accuracy achieved on both datasets, with the better of the two results emphasized. The approaches are presented in the order of the best accuracy they showed in our experiments. The table also shows the accuracy that the authors reported in their corresponding papers.

**Table 1:** Quantitative comparison of multilingual sentiment analysis approaches

Paper	Approach	Machine learning techniques	Reported accuracy (%)	Accuracy in our tests	
				Movie reviews (%)	Product reviews (%)
Singh et al. [52]	SentiWordNet	NB, SVM	81.14	**71.28**	65
Shi and Li [47]	Supervised machine learning	SVM	85	**69.40**	68
Boiy and Moens [10]	Machine learning	SVM, MNB, MaxEnt	86.35	**67.40**	65
Tan and Zhang [54]	Feature selection techniques such as document frequency, Chi-square, mutual information, and information gain	SVM, NB, K-nearest neighbour classifier, Winnow classifier	82	62	**65.24**
Al-Ayyoub et al. [3]	Lexicon-based	SVM	86.89	61	**64**
Balahur and Turchi [5]	Hybrid + SVM SMO	Hybrid, SVM SMO	69.09	62	**63**
Mahyoub et al. [30]	Lexicon-based	SVM	96	61	**62**
Zagibalov and Carroll [71]	Seed-word selection	SVM	81	61	**62**
Zhu et al. [73]	Bootstrapping	SVM	62.09	57	**59.90**
Habernal et al. [23]	Supervised machine learning	SVM, MaxEnt	64	**59.75**	58
Mizumoto et al. [34]	Bootstrapping	Bootstrapping	45	**42**	41

Bold values indicate best performance

Performance comparison of state-of-the-art approaches shows a difference between the accuracy reported by the respective authors and the accuracy obtained in our experiments. We attribute this mainly to the lack of detail in the original publications, which did not allow for exact reproduction of the techniques in our implementation.

In some cases, the reported results are not comparable with our results because we used different experiment settings, tools, and datasets. For example, Boiy and Moens [10] reported 86.35 % accuracy, but we obtained 67.40 %; Habernal et al. [23] reported 64 % accuracy, but we obtained 59.75 %. Researchers used different datasets, such as the stock market, movie reviews, product reviews, hotel reviews, and tweets. Tan and Zhang [54] used an online reviews dataset to evaluate the performance of their approach, while we used product reviews, i.e. the Blitzer dataset; Shi and Li [47] used a hotel reviews dataset, while we used movie reviews, i.e. the Cornell movie review dataset.

In addition, we employed different linguistic resources. For example, Singh et al. [52] used SentiWordNet, and Mahyoub et al. [30] and Al-Ayyoub et al. [3] used Arabic linguistic resources, while we used SentiWordNet. Some of these approaches listed here were developed for languages other than English. For example, Tan and Zhang [54] developed their approach for sentiment analysis of Chinese texts, and Habernal et al. [23] for sentiment analysis in Czech. We used an English dataset to evaluate the performance of these approaches. Further, the state-of-the-art approaches employed different tools to build machine learning classifiers, such as SVM^Light^, WEKA, and LibSVM, while we employed LibSVM and Weka for our experiments.

In our experiments, the approach by Singh et al. [52] showed the best accuracy. Our experiments also suggest that the SVM classifier usually outperforms by a large margin all other classifiers.

### Qualitative Comparison

Different researchers used different experimental settings. Tan and Zhang [54] selected traditional features such as document frequency, information gain, mutual information, and Chi-square test, while Habernal et al. [23] used n-grams, emoticons, and part-of-speech features. Some of these features include multiword expressions, which suffer from the data sparsity problem. Due to this, such features are not effective and contain a large amount of noise [65]. Syntactic n-grams have performed better than traditional linear n-grams because they are more informative and less arbitrary. These features are also more accurate in comparison with information gain, Chi-square test, and n-grams [1, 48, 50].

The approach proposed by Singh et al. [52] obtained good accuracy, though it requires extensive calculation of many PMI values, which is computationally expensive. The approach proposed by Mizumoto et al. [34] is only applicable to stock market news; it showed very low accuracy with other types of datasets such as movie reviews or product reviews.

The sentiment analysis approaches have different advantages and disadvantages. Table 2 summarizes the advantages and disadvantages of different approaches.

**Table 2:** Qualitative comparison of multilingual sentiment analysis approaches

Method	Languages	Advantages	Disadvantages
Shi and Li [47]	English	Very simple to implement	Feature selection is ineffective
Boiy and Moens [10]	English	Can be easily extended to other languages	Computationally expensive
Singh et al. [52]	English	Useful for both small and large datasets	Computationally expensive: heavy PMI calculation
Mizumoto et al. [34]	English	Automatically produces a dictionary for stock market sentiment analysis	Only applicable to stock market sentiment analysis
Habernal et al. [23]	Czech	Large Czech dataset created, which can be used for other researchers	Only applicable to Czech sentiment analysis; needs further development
Tan and Zhang [54]	Chinese	Various feature selection techniques such as information gain, Chi-square test, mutual information, and document frequency	Requires more trained data
Zagibalov and Carroll [71]	Chinese	Can be extended to multilingual sentiment analysis	Computationally expensive
Balahur and Turchi [5]	English, French, Italian, German, Spanish	Can be used for more than one language	No resources available for multilingual sentiment analysis
Zhu et al. [73]	Chinese	Effective feature selection	Requires very large dataset
Mahyoub et al. [30]	Arabic	Proposed Arabic SentiWordNet	Cannot handle informal words
Al-Ayyoub et al. [3]	Arabic	Proposed Arabic linguistic tools	Cannot handle different dialects
Ghorbel and Jacot [21]	French	Good precision; one of few works on French	Need in translation affects precision

## Conclusions

We gave an overview of state-of-the-art multilingual sentiment analysis methods. We described data pre-processing, typical features, and the main resources used for multilingual sentiment analysis. Then, we discussed different approaches applied by their authors to English and other languages. We have classified these approaches into corpus-based, lexicon-based, and hybrid ones.

The real value of technique for the research community corresponds to the results that can be reproduced with it, not in the results its original authors reportedly obtained with it. To evaluate this real value, we have implemented eleven approaches as closely as we could basing on their descriptions in the original papers, and tested them on the same two corpora. In the majority of the cases, we obtained lower results than those reported by their corresponding authors. We attribute this mainly to the incompleteness of their descriptions in the original papers. In some cases, though, the methods were developed for a specific domain, so in such cases comparison on our test corpora may not be fair. A lesson learnt was that for a method to be useful for the research community, authors should provide sufficient detail to allow its correct implementation by the reader.

According to our results, the approach proposed by Singh et al. [52] outperforms other approaches. However, this approach is computationally expensive and has been tested only on English-language data. The least accurate approaches of those that we considered were the ones proposed by Zhu et al. [73], Habernal et al. [23], and Mizumoto et al. [34].

The main problem of multilingual sentiment analysis is the lack of lexical resources [18]. In our future work, we are planning to develop a multilingual corpus, which will include Persian, Arabic, Turkish, and English data, and compare different methods by applying them to this corpus.
